# Minimally invasive reconstruction of acute acromioclavicular joint injuries using the TwinBridge button system

**DOI:** 10.1007/s00590-022-03293-0

**Published:** 2022-05-31

**Authors:** Maurice Balke, Arasch Wafaisade, Juergen Hoeher, Oliver Greshake

**Affiliations:** 1 Sportsclinic Cologne, University of Witten/Herdecke, Ostmerheimer Str. 200, 51109 Cologne, Germany; 2 Department of Trauma and Orthopedic Surgery, Cologne-Merheim Medical Center, University of Witten/Herdecke, Cologne, Germany

**Keywords:** Shoulder, Acromioclavicular, AC joint, Shoulder stabilization

## Abstract

**Introduction:**

Acute acromioclavicular joint (ACJ) injuries are among the most common shoulder injuries in active young adults. The most frequently used surgical treatments include the hook plate implantation and arthroscopic treatment using flip-button systems. The aim of this study was to evaluate the results of treating acute ACJ injuries using a new minimally invasive implant based on a flip-button system.

**Material and methods:**

From January 2016 to October 2019, a total of 20 patients with acute ACJ injuries (1 × Type III, 3 × Type IV, 16 × Type V) underwent surgery using the Twinbridge implant (Smith & Nephew). It is a prefabricated construct consisting of two Endobuttons connected with an UltraTape. One button is placed under the coracoid using a special aiming device and two buttons are placed on the clavicle. Preoperatively, 1 day postoperatively, 3 months and at least 1 year postoperatively, patients were clinically examined and bilateral stress view and axial radiographs were obtained. At final follow-up, the simple shoulder test (SST), Taft score, Constant score, and ACJ instability (ACJI) score were recorded and a side-to-side ratio of the coracoclavicular (CC) distance was calculated.

**Results:**

All 20 patients were contacted at final follow-up at a mean of 28 (min. 13, max 50) months. Six patients were not willing to come for a clinical and radiographic examination and were contacted via telephone. All six patients were free of complaints. Another two patients free of complaints refused radiographs at final follow-up. The patients presented a mean SST of 99.6% (20 patients, min. 91.7, max. 100), Taft score of 11.6/12 points (12 patients, min. 10, max. 12), ACJI of 85.5/90 points (12 patients, min. 78, max. 90), and a Constant score of 97.1 (14 patients, min. 81.0, max. 100) for the affected shoulder. Preoperative stress view images revealed a mean side-to-side difference of the CC distance with a ratio of 1:2.34 (min. 1:1.80, max. 1:3.33). At final follow-up, CC distance was calculated with a mean ratio of 1:1.12 (min. 1.1, max. 1:1.38). Axial images showed a proper position in all cases. A “perfect” radiological result was achieved in six patients (50%) with a side-to-side CC distance of less than 10% (ratio 1:1.1 or less). A Rockwood type II result was achieved in five patients (42%) with a distance of 10 to 25% (ratio 1.11–1.25). One (8%) presented with a Rockwood type III result with a difference of more than 25% (ratio 1:1.38) and was considered a radiological failure.

**Conclusions:**

When used correctly, the Twinbridge implant offers good-to-excellent clinical and radiographic results using a minimally invasive surgical technique. Complication rate is comparable to other button-systems.

## Introduction

Sprains or dislocations of the acromioclavicular joint (ACJ) account for 12% of all shoulder injuries and are very common injuries among active, mostly male, adults [27]. In 2013, Beitzel et al. [6] published a current concepts review summarizing 151 different surgical techniques for ACJ reconstruction. Of the several techniques described [10, 12, 16], in recent years, the hook plates minimally invasive technique or arthroscopic techniques using button-systems have gained popularity [2, 3, 5, 14, 19].

Major disadvantages of the hook plate are the need for implant removal and the potential loss of correction after removal [8, 9, 16, 26]. In contrast to the hook plate, arthroscopic implantation of button-systems is technically demanding and thus mostly performed by specialized shoulder surgeons [3]. The reported clinical results after arthroscopic ACJ stabilization were excellent but radiologically a significant side-to-side difference in the coraco-clavicular distance was observed due to a loss of reduction [11, 21, 22]. Petersen et al. published in 2010 a suture-button technique called minimally invasive ACJ reconstruction (MINAR—Karl Storz, Tuttlingen, Germany) which is performed in a straightforward open approach without the need for arthroscopy [17], where one button is placed under the coracoid process and one on top of the clavicle. This technique could also be used by less specialized surgeons and the published clinical results were excellent [15, 20]. However, the reported complication rate was 10.8–14.3%, and radiologically, there still was a significant loss of reduction [4, 20, 23].

Here, we publish the results of a comparable button-technique using a flip-button implant called the Twinbridge (Smith & Nephew). One button is placed under the coracoid process and two buttons are placed on top of the clavicle. The procedure can either be performed in a minimally invasive manner or arthroscopically.

The hypothesis was that the clinical and radiological results are at least comparable to the published results for similar techniques.

## Material and methods

From January 2016 to October 2019, a total of 20 consecutive patients with acute ACJ injuries (1 × Type III, 3 × Type IV, 16 × Type V according to Rockwood [18]) were operated on with the Twinbridge implant (Smith & Nephew). Eighteen surgeries were performed by the first author, two by the senior author, both specialized and experienced shoulder surgeons. All surgeries were done within 2 weeks of the trauma. Preoperatively, directly postoperatively, 3 months and at least 1 year postoperatively, patients were clinically examined and stress view and axial radiographs were obtained. At the final follow-up, the range of motion, simple shoulder test (SST), Taft score, Constant score, and ACJ instability (ACJI [22]) score were recorded. All follow-up examinations were performed by the first author.

### Surgical Technique

**Fig. 1:**
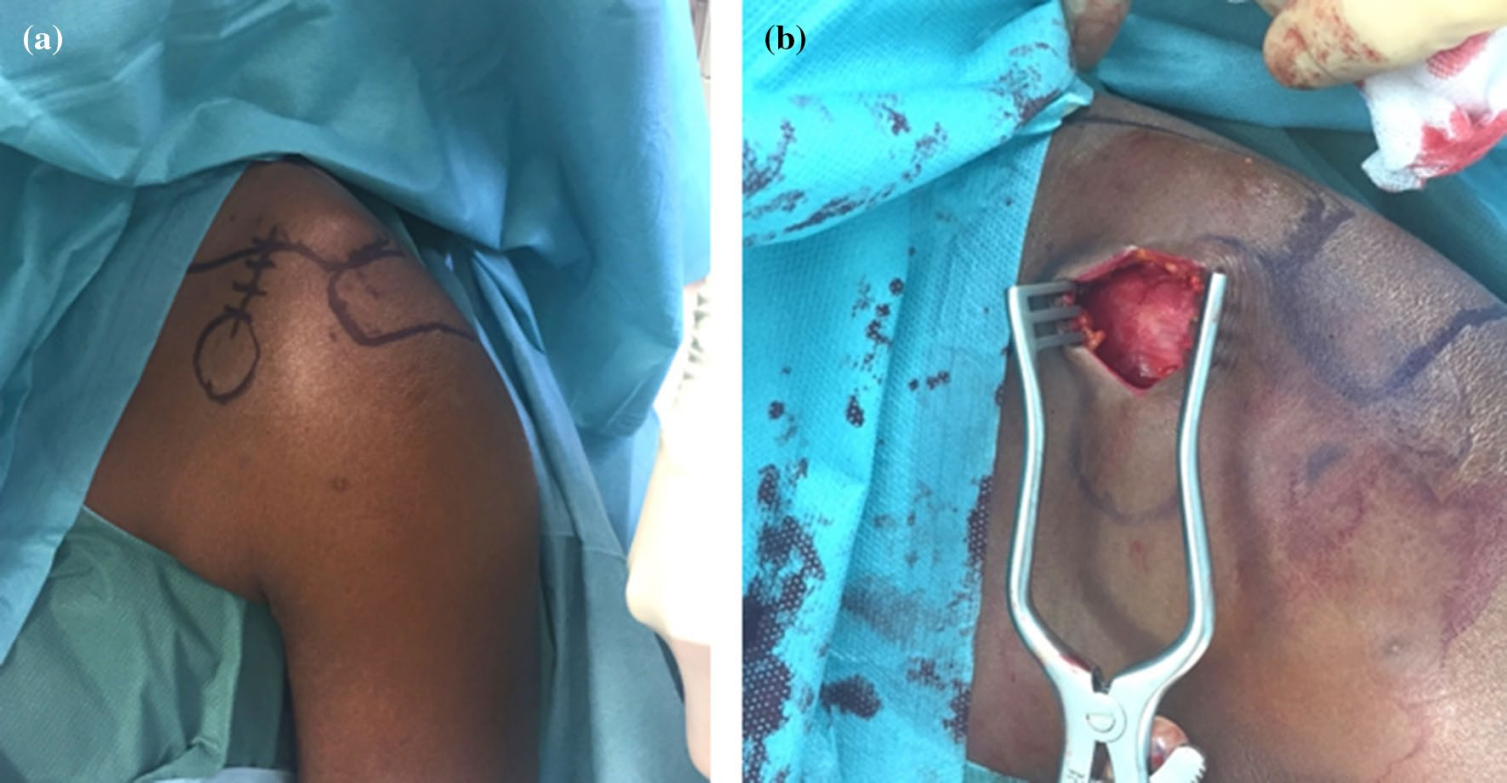
**A** + **B** Anatomical landmarks and skin incision

All surgeries were carried out in the beach chair position. After drawing of anatomic landmarks, a sabre cut incision was made approximately 3 cm medial to the AC joint (Fig. 1 A + B).

**Fig. 2:**
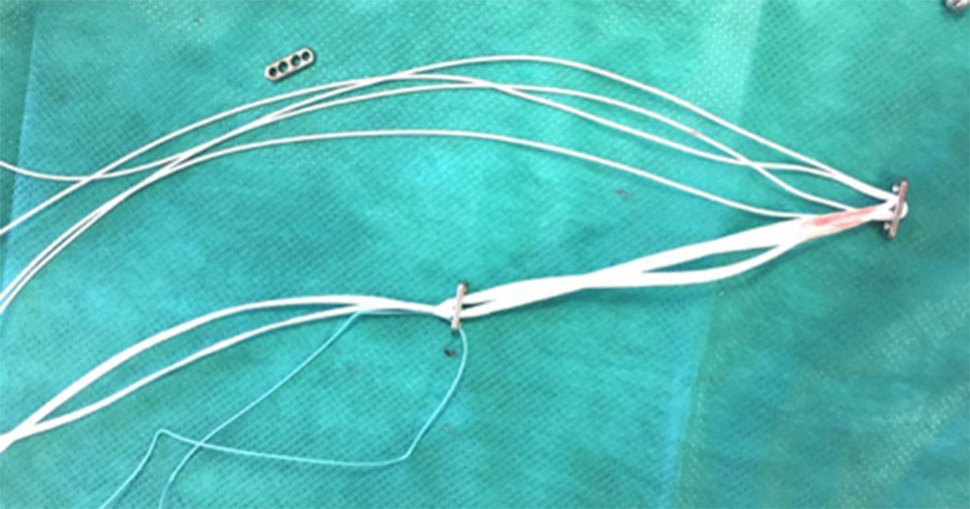
Modified Twinbridge implant with ultratape and polyester sutures

The underlying fascia was incised perpendicular to the skin incision in a longitudinal direction. The anterior border of the lateral clavicle was exposed, and the tissue was prepared down to the coracoid process. The fascia lateral to the coracoid process was incised and spread to a size of approximately 1 cm. The aiming device was inserted and its hook was positioned under the coracoid process. The drill guide was inserted, and a 2.4 mm K-wire was drilled through the bone. The drill guide was removed but the K-wire left in place. The correct position in the center of the coracoid process was verified manually and if correct the wire was overdrilled with a 4.5 mm drill. If in doubt, the correct position of the K-wire in the center of the coracoid was verified by fluoroscopy. The Twinbridge implant (Fa. Smith & Nephew), a prefabricated construct consisting of two endobuttons connected by an ultratape, was modified by changing the two sutures (UPS 5 polyester) originally inserted for flipping the distal button to the central holes of the distal endobutton (the area where the ultratape runs through) but not through the proximal button (Fig. 2).

The distal button was brought into the drill hole in the coracoid process using a needle holder and pushed through the hole using the stump side of the K-wire. The button was flipped by pulling back all tapes and sutures.

**Fig. 3:**
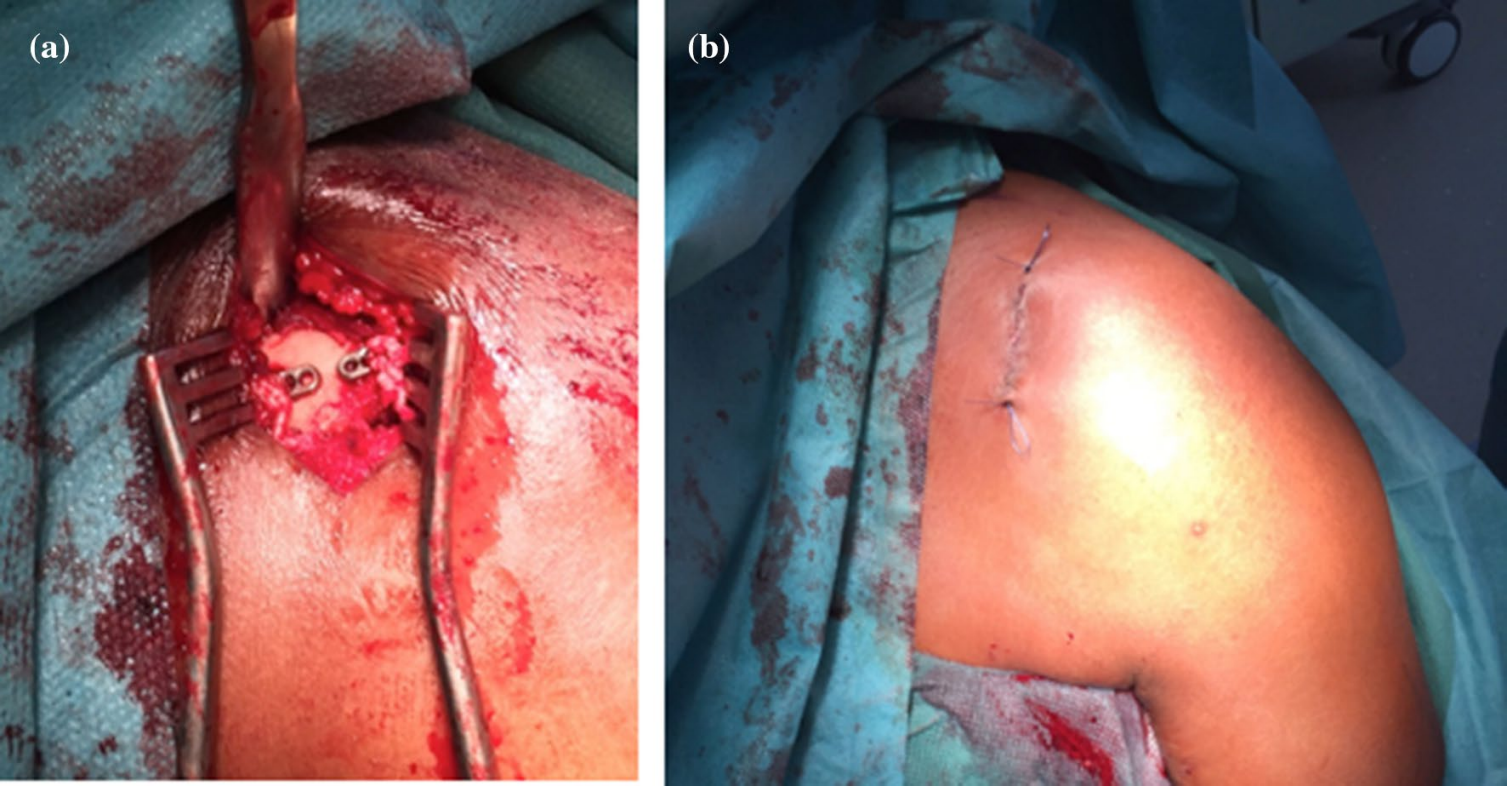
**A** + **B** Endobuttons on clavicle before closure of fascia and skin

Another drill hole was made in the lateral clavicle approximately 4.5 cm medial to the AC joint using the 4.5 mm drill and a second hole approximately 1.5 cm lateral to the first was made using only the K-wire. Then, by using shuttle sutures inserted through the holes with help of a spinal needle, the proximal button loaded with the ultratape was pulled through the medial drill hole and the four free sutures were pulled through the lateral drill hole. The clavicle was reduced under visual and radiographic control, and the zip loop construct of the ultratape was tied over the button on the upper side of the clavicle. Another button was loaded with the four free sutures (2 pairs) which were then tied over the second lateral button (Fig. 3A). Care was taken to fully close the trapezoidal and deltoid fascia, subcutaneous tissue and skin (Fig. 3B).

Postoperative treatment included recommendation to wear a sling for 3 weeks and restrict range of motion to 90° of abduction and anteversion for the first 6 weeks. From the 7th week, full range of motion was allowed under guidance of a physiotherapist with emphasis on the scapulothoracic motion. Contact sports and full return to activities were allowed 4 months after surgery.

### Radiological follow-up

Preoperatively, directly postoperatively, 3 months and at least 1 year postoperatively, patients were clinically examined, and bilateral stress view and axial radiographs were obtained. Preoperatively, 3 months post-surgery and at final follow-up, stress radiographs were acquired with the patient carrying a 10 kg kettlebell in each hand. Radiographs immediately (one day) after surgery were taken without weight.

On each axial radiographs, the horizontal position of the lateral clavicle was evaluated, and on the bilateral stress views, the coracoid-clavicular (CC) distance was measured using Horos for Macintosh. Since radiographs were done without a calibrated measuring device, the ratio of the CC distance of the affected to the healthy side was calculated. These values were used to classify the injury severity according to Rockwood and to calculate the ACJI [22] at final follow-up. All measurements were done by the first author.

A side-to-side difference at final follow-up was classified as “perfect” if it was less than 10% (1:1.1), Rockwood type II if 10 to 25% (1:1.11–1:1.25), Rockwood type III if 26–100% (1:1.26–1:1.9), and Rockwood type V if it was more than 100% (1:2.0).

Informed consent was obtained from all patients. The study was approved by the local ethical committee (blinded for review 28/2018).

### Statistical analysis

Statistical analysis was performed using Microsoft Excel 16.12.27. A p value < 0.05 was considered significant (paired t test).

## Results

Mean age of patients at time of injury was 37 (min. 17, max. 65) years. Seventeen patients were male and three patients were female. Six injuries happened during a fall in cycling, five while playing soccer, two each in Judo, equestrian, motorcycling, and skiing/snowboarding, and one while walking.

Mean time between trauma and surgery was 6.7 (min. 1, max. 12) days.

All 20 patients were contacted at final follow-up at a mean of 28 (min. 13, max, 50) months. Six patients were not willing to come for a clinical and radiographic examination and were only contacted via telephone. All six patients were free of complaints and had unrestricted range of motion. Another two patients refused the radiographic examination at final follow-up. These two were also free of complaints and had unrestricted range of motion. Thus, 12 patients completed all final examinations including radiographs, and 14 were available for clinical examination. These patients presented a mean SST of 99.6% (20 patients, min. 91.7, max. 100), Taft score of 11.6/12 points (12 patients, min. 10, max. 12), ACJI of 85.5/90 points (12 patients, min. 78, max. 90), and a Constant score of 97.1 (14 patients, min. 81.0, max. 100) for the affected shoulder. The contralateral side showed a mean SST of 99.6% (min. 91.7, max. 100), Taft score of 11.9/12 points (min. 11, max. 12), ACJI of 89.6/90 points (min. 85, max. 90), and a Constant score of 99.9 (min. 99, max. 100). Clinical evaluation of anteroposterior stability of the lateral clavicle was routinely done at last follow-up and did not show any instability compared to the contralateral side.

Statistically significant differences (affected vs. contralateral shoulder) were only found for the ACJI (*p* = 0.009).

### Radiological outcome

**Fig. 4:**
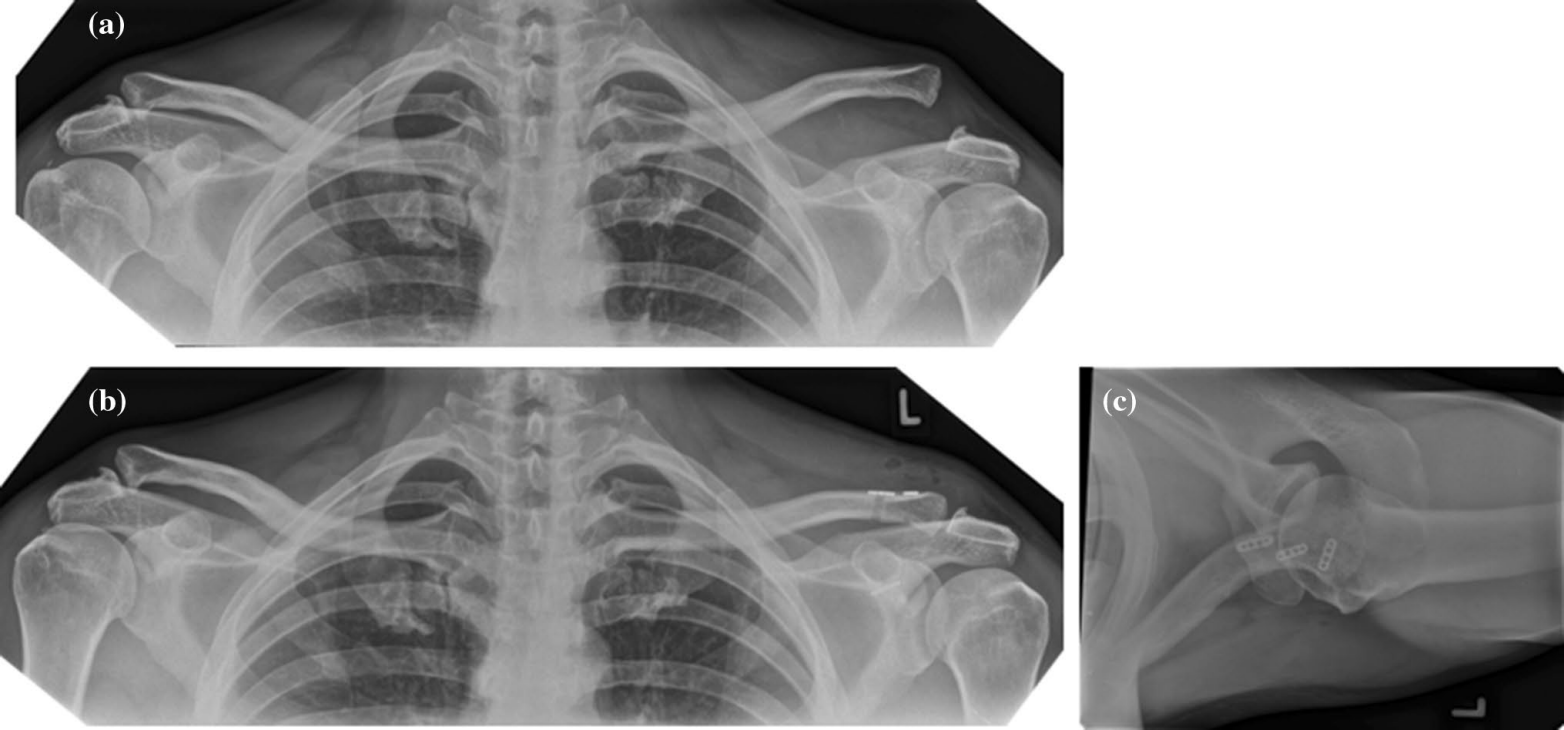
**A**, **B**, **C** Stress radiographs before and after reconstruction and axial view confirming horizontal alignment

The preoperative stress view images (example see Fig. 4A) revealed a mean side-to-side difference of the CC distance with a ratio of 1:2.34 (min. 1.80, max. 3.33).

Sixteen were classified as grade V injuries (ratio 1:2–1:3.33), three as grade IV (ratio 1:1.86–1:1.89), and one as grade III (ratio 1:1.89) according to Rockwood. On the 1 day postoperative radiograph (bilateral stress view without weight + axial), nine showed an overcorrection of a mean ratio of 1:1.45 (min. 1:1.13, max. 1:2.3). In eight without overcorrection, the mean ratio was 1:1.32 (min. 1:1, max. 1:1.83). The postoperative radiograph was not available for three patients. In all cases, the axial images showed good horizontal alignment.

In bilateral stress views with 10 kg weight three months after surgery, two showed an overcorrection of 1:1.33 and 1:1.11. Thirteen showed a correction to a mean ratio of 1:1.14 (min. 1:1, max. 1:1.38). In five cases, radiographs were not available.

At final follow-up (Fig. 4B), CC distance was calculated for 12 patients with a mean ratio of 1:1.12 (min. 1:1, max. 1:1.38). Axial images showed a proper position in all cases (Fig. 4C). None showed an overcorrection, and eight patients did not have radiographs. Thus, a “perfect” result was achieved in six patients (50%) with a side-to-side CC distance of less than 10% (ratio 1:1.1 or less). A Rockwood type II result was achieved in five patients (42%) with a distance of 10–25% (ratio 1.11–1.25). One (8%) presented with a Rockwood type III result with a difference of more than 25% (ratio 1:1.38) and was considered a radiological failure. Clinically, she was free of complaints and fully returned to sports. Thus, 92% showed a perfect or good radiological result at final follow-up.

### Complications

In one patient, the coracoid button was placed too laterally, leading to a dislocation of the button. At final follow-up (36 months post-surgery), the ratio of the side-to-side difference of the CC distance was found to be 1:1.13 and the patient was symptom-free. One patient developed a pneumothorax 5 days postoperatively, probably due to surgery, because in this particular case, the aiming device was not used. She was also free of complaints but was the only patient with a SST less than 100 (91.7 points). Two patients showed ossifications and migration of the medial button into the clavicle without clinical consequences. One patient presented with a fracture of the coracoid 6 months after surgery, which was treated conservatively. At final follow-up of 15 months, he was free of complaints with a CC distance of a ratio of 1:1.09.

No patient was reoperated during the study period.

## Discussion

The most important finding of the present study is that minimally invasive AC-joint reconstruction using the Twinbridge construct leads to good-to-excellent clinical results. At least one year after surgery, shoulder function was found to be comparable to the contralateral shoulder. Ninety-two percent presented with excellent to good radiological results with loss of reduction of less than 25%.

A comparable technique was published by Rosslenbroich et al. [19, 20]. They investigated 83 patients treated with a minimally invasive AC-joint reconstruction (MINAR). This system also uses a subcoracoidal flip-button, but only one clavicular button [20]. Despite excellent clinical results with a mean Constant score of 94.7, the revision rate with nine patients (10.8%) was high. Of them, eight experienced a recurrent dislocation and 21.6% showed radiological failure (loss of reduction of more than 50%) at mean follow-up of 39 months. CC distances were not measured, and radiological failure did not correlate with clinical results. Recently, Banerjee et al. also published a high rate of radiological failure despite excellent clinical results of the MINAR system [4]. The study included 45 patients with a mean follow-up of 25.3 months. Despite excellent clinical results and a high patient satisfaction rate, only 14 patients (31.1%) had a perfect reduction in the vertical plane as measured by a CC distance of up to 10% compared to the contralateral side.

In a current-concepts review, Gowd et al. published an overall failure rate of 18.3% on average of button techniques for AC-joint reconstructions [12]. Most commonly used was the arthroscopic TightRope Technique (Arthrex, Naples, FL/USA) [3] with a failure rate of 20.5% [12]. However, it has to be admitted that there is no clear definition of failure. Respective definitions might differ and include loss-of reduction, CC distance of more than 50% or of more than 25% [12, 24, 25].

As published by Schliemann et al. [23], malpositioning of the coracoid tunnel and subsequent button dislocation might be the main reason for failure. In their study, 9 out of 63 patients (14.3%) had revision surgery of which eight had recurrent instability. The coracoid tunnel was placed too far lateral in six cases and too far anterior in two cases, one patient had a coracoid fracture. Although the mean difference of the CC distance was only 1.4 mm, five patients presented with a complete loss of reduction and recurrent Rockwood V injuries. Positioning of the coracoid tunnel too far laterally and a coracoid fracture was also observed in our study.

Another possible explanation of the high radiological failure rate might be the use of only one clavicular button and the non-absorbable suture in the MINAR system. However, results of a modified technique with one coracoidal and two clavicular buttons published by Breuer et al. [7] showed a loss of reduction of 2.1 mm side-to-side difference of the CC distance. In their study, 59% of the patients did not have any loss of reduction. The technique described by Breuer et al. is comparable to the technique presented here. Although their study included 65 patients, the results are comparable. Hence, we decided to use two buttons on the clavicula.

Several studies focused on the arthroscopic TigthRope (Fa. Arthrex) technique. Good results were reported by Glanzmann et al. with a minimum follow-up of 24 months after double TightRope stabilization [11]. The mean CC distance was 2 mm in 19 patients with Rockwood III or IV injuries. A failure was defined as a CC distance of more than 2 mm and happened in six patients. Salzmann et al. [21] reported good clinical results after a mean follow-up of 30.6 months in 23 patients but unsatisfactory AC joint alignment in eight cases either in the coronal, axillary, or both planes. However, the clinical results did not correlate with the radiological alignment. The mean CC distance reported by Scheibel et al. [22] after arthroscopic double TightRope reconstruction was 4.2 mm. Twenty-eight patients with only high-grade Rockwood V injuries were evaluated at a mean follow-up of 26.5 months. Radiologically 42.9% presented signs of posterior instability and had significantly inferior Taft and ACJI scores. These findings support the theory that in arthroscopic ACJ reconstruction, an additional horizontal cerclage might reduce the risk of persistent posterior translation [13]. However, there is still no clear evidence of its superiority over CC reconstruction [1]. It should be mentioned that the implant presented here could also be used arthroscopically.

Several limitations of the present study have to be mentioned. The sample size with 20 patients was relatively low and some patients were not willing to come for follow-up examinations. Only 12 patients underwent the last follow-up radiographic control. Radiographs were not scaled to reliably measure the CC distance. Thus, a ratio, independent of possible magnifications was calculated. The ACJI score was calculated using axial radiographs to evaluate horizontal stability, although it was originally described using Alexander views. These are more suitable to detect dynamic horizontal instability.

## Conclusions

When used correctly, the Twinbridge implant offers good-to-excellent clinical and radiographic results using a minimally invasive surgical technique. Complication rate is comparable to other button-systems.
